# “True” Lateral Imaging for Lumbar Radiofrequency Medial Branch Neurotomy

**DOI:** 10.1093/pm/pnz313

**Published:** 2019-11-21

**Authors:** Patrick H Waring

**Affiliations:** Pain Intervention Center, Metairie, Louisiana, USA

The use of thermal radiofrequency (RF) energy to destroy medial branch nerves innervating specific lumbar facet joints for the palliative relief of chronic low back pain has been described extensively [1]. Precise procedural technique is a critical element of successful thermal RF neurotomy [2,3]. Ideally, the distal tip of the RF cannula should be placed in close proximity and parallel to the course of the medial branch nerve for maximal denervation.

**Figure 1:**
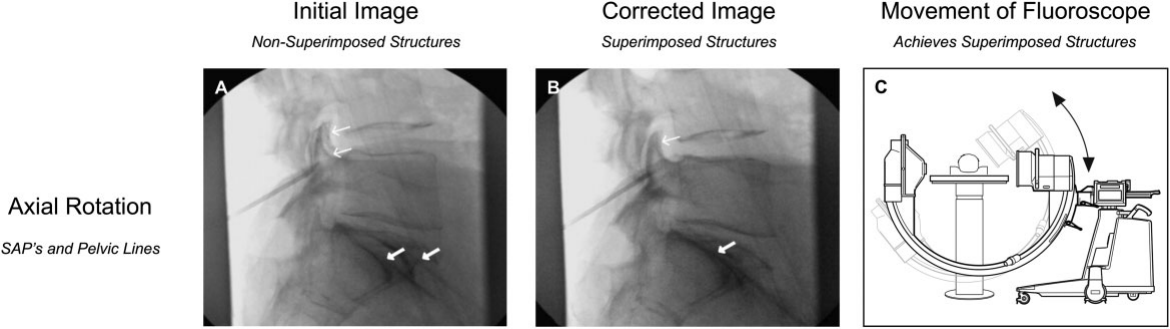
Axial fluoroscopic rotation in the conduct of L4 medial branch neurotomy lateral imaging. A) Thin arrows indicate nonsuperimposed anterior aspects of L5 superior articular processes (SAPs), and thick arrows indicate nonsuperimposed pelvic lines. B) After axial rotation, the thin arrow indicates superimposed anterior aspects of L5 SAPs, and the thick arrow indicates superimposed pelvic lines.

**Figure 2:**
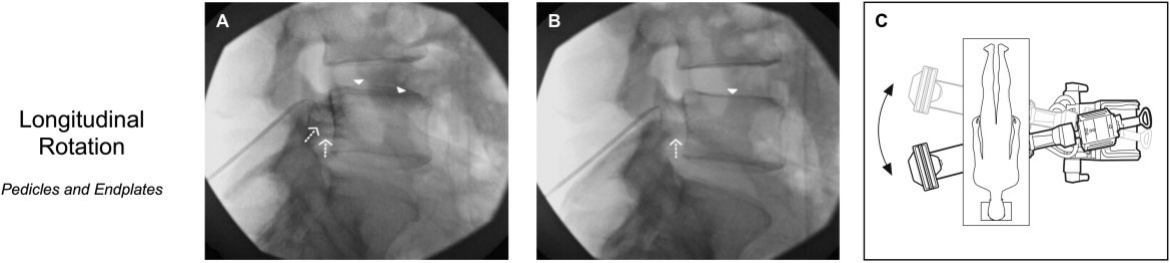
Longitudinal fluoroscopic rotation in the conduct of L4 medial branch neurotomy lateral imaging. A) Dashed arrows indicate nonsuperimposed inferior aspects of L5 pedicles, and arrowheads indicate the nonsuperimposed L5 superior endplate. B) After longitudinal rotation, the dashed arrow indicates superimposed inferior aspects of the pedicles, and the arrowhead indicates the superimposed superior endplate at the L5 level.

A “true” lateral fluoroscopic imaging technique has been proposed for confirmation of correct cannula placement before initiation of destructive thermal energy [4]. Each lumbar segment’s “true” lateral image can easily be obtained by fluoroscopic manipulation to superimpose four readily identifiable radiographic features. Two features (anterior aspects of superior articular processes and pelvic lines) are superimposed by axial rotation of the lateral fluoroscope (Figure 1), and two features (pedicles and endplates) are superimposed by longitudinal rotation (Figure 2).

The use of the proposed features for “true” lateral lumbar imaging may enhance thermal RF lumbar medial branch neurotomy technique, as the final position of the RF cannula in relation to its target structures is more accurately visualized at each segment.
